# Overview of methods and available tools used in complex brain disorders

**DOI:** 10.12688/openreseurope.16244.1

**Published:** 2023-09-19

**Authors:** Loukas Ilias, George Doukas, Michael Kontoulis, Konstantinos Alexakis, Ariadni Michalitsi-Psarrou, Christos Ntanos, Dimitris Askounis

**Affiliations:** 1 Decision Support Systems Laboratory, School of Electrical and Computer Engineering, National Technical University of Athens, Athens, 15773, Greece

**Keywords:** neurocognitive disorders, sleep disorders, epilepsy, data analytics, machine learning, data analytics tools

## Abstract

Complex brain disorders, including Alzheimer’s dementia, sleep disorders, and epilepsy, are chronic conditions that have high prevalence individually and in combination, increasing mortality risk, and contributing to the socioeconomic burden of patients, their families and, their communities at large. Although some literature reviews have been conducted mentioning the available methods and tools used for supporting the diagnosis of complex brain disorders and processing different files, there are still limitations. Specifically, these research works have focused primarily on one single brain disorder, i.e., sleep disorders or dementia or epilepsy. Additionally, existing research initiatives mentioning some tools, focus mainly on one single type of data, i.e., electroencephalography (EEG) signals or actigraphies or Magnetic Resonance Imaging, and so on. To tackle the aforementioned limitations, this is the first study conducting a comprehensive literature review of the available methods used for supporting the diagnosis of multiple complex brain disorders, i.e., Alzheimer’s dementia, sleep disorders, epilepsy. Also, to the best of our knowledge, we present the first study conducting a comprehensive literature review of all the available tools, which can be exploited for processing multiple types of data, including EEG, actigraphies, and MRIs, and receiving valuable forms of information which can be used for differentiating people in a healthy control group and patients suffering from complex brain disorders. Additionally, the present study highlights both the benefits and limitations of the existing available tools.

## 1 Introduction

The Multidisciplinary Expert System for the Assessment & Management of Complex Brain Disorders (MES-CoBraD) is an interdisciplinary project combining real-world data (RWD) from multiple clinical and consumer sources towards improving diagnostic accuracy and therapeutic outcomes in people with complex brain disorders (CoBraD), as reflected in neurocognitive (dementia), sleep, and seizure (epilepsy) disorders and their interdependence. CoBraD, as represented in neurocognitive (e.g., Alzheimer’s dementia), sleep, and seizure (i.e., epilepsy) disorders, are chronic conditions that have high prevalence individually and in combination, leading to disability that interferes with activities of daily living (ADLs) and worsens quality of life. According to the World Health Organization (WHO), more than 55 million people have dementia worldwide, while every year there are nearly 10 million new cases. Similarly, WHO states that around 50 million people worldwide have epilepsy. According to estimates, 50 to 70 million people in the U.S. have ongoing sleep disorders ( www.sleepfoundation.org).

The MES-CoBraD project exploits real-world data, including questionnaires / structured examinations, neuropsychological tests, imaging data, electroencephalogram (EEG)/polysomnography (PSG), biosamples of blood and cerebrospinal fluid biomarkers, medical device data, and consumer technology data. Several methods have been proposed using the aforementioned data for supporting the diagnosis of complex brain disorders. At the same time, many tools have been introduced which process these data to extract valuable forms of information.

Our main contributions are two-fold. Specifically, we present the first review study, which presents the most common methods used for processing different kinds of data, including PSG, magnetic resonance imaging (MRI), actigraphies, targeted at supporting the diagnosis of multiple complex brain disorders. On the contrary, other research works focus either on one type of data or one single brain disorder. Next, to the best of our knowledge this is the first study listing all the available tools (along with the benefits and drawbacks per tool), which can be used for supporting the diagnosis of complex brain disorders.

This paper is organized as follows: Section 2 presents the related work. Specifically, in Section 2.1, we mention some existing research initiatives using PSG signals along with data analytics and machine learning techniques for supporting the diagnosis of neurocognitive, epilepsy, and sleep disorders. Section 2.2 and Section 2.3 present the research conducted in terms of actigraphies and MRI respectively. In Section 3, we mention some tools, which can be used for performing experiments using all the aforementioned data, namely PSG, MRIs, actigraphies, questionnaires, and so on. Finally, concluding remarks are provided in Section 4.

## 2 Related work

### 2.1 Polysomnography (PSG)

EEG was first recorded on the animal brain in 1875 by Richard Caton and on the human brain in 1929 by Hans Berger ^1^. The EEG signal displays the brain’s electrical activity as recorded by placing metal electrodes over the scalp. A 10–20 electrode placement system has been devised by the International Federation of Societies for Electroencephalography to specify the location of the electrodes with respect to the underlying area of the cerebral cortex. The neuronal activities are measured as electric currents generated by the synchronized activity of a group of specialized pyramidal cells inside the brain. Voltage variations observed in the EEG measurements demonstrate the occurrence of neuronal activity.

EEG signals are non-linear and non-stationary in nature ^2, 3^. Abnormal brain activity can be distinguished without difficulty from a normal one using signal processing methods since EEG traces tend to differ. Therefore, in clinical practice, EEG signals can be used as a reliable, non-invasive indicator for the observation of mental states and the diagnosis and treatment of brain disorders. EEG signals, with the support of computer-aided technologies, assist in diagnosing various neurological and neuropsychiatric disorders, such as epilepsy ^4– 7^, dementia ^8, 9^, depression ^10– 13^, alcoholism ^14– 16^, sleep disorders ^17, 18^, concussions ^19^, strokes ^20^, and problems associated with trauma ^21, 22^.

EEG signals may indicate the following brain rhythms: delta (0.1–3.5) waves, theta (4–7.5 Hz) waves, alpha (8–13 Hz) waves, beta (14–30Hz) and gamma (>30 Hz) waves. Brain rhythms are related to occurrence regions and specific characteristics, and therefore support inferring a patient’s mental state ^23^. Any small changes in these waves’ patterns support the identification of neuronal activity or the diagnosis of neurological disorders.

Despite the significance of EEG signals, it is difficult and time-consuming to get useful information just by observation. In this context, machine learning approaches are increasingly being applied in EEG signal processing and analysis providing preliminary results to neurologists for further assessment. The processing and analysis of EEG signals consist of specific steps ^3, 24– 26^, as the academic literature suggests, as follows: signal acquisition, signal preprocessing and enhancement, feature extraction, feature selection or dimensionality reduction and finally machine learning training and testing and results analysis. Signal acquisition methods should ensure the quality of the acquired data, while publicly available EEG datasets, specifying the devices used for the collection, the sampling rate and the number of subjects participating, are frequently used in research studies. The second step, signal preprocessing and enhancement involves the removal of noise and artefacts from the signals. Artefacts may generally fall into two classes, based on their origin ^27^: technical artefacts, which arise from external, technical issues due to the data collection process, such as power line interferences, and physiological artefacts, which are generated by the human body, such as eye blinks, eye

movements (EOG artefacts), muscle activities (EMG artefacts), and heartbeat (ECG artefacts). Various artefact removal methods have been proposed for the handling of all these artefacts. Some of the most common ones encountered in the literature are independent component analysis (ICA), principal component analysis (PCA), common average referencing (CAR), common spatial patterns (CSP), surface Laplacian (SL), adaptive filtering and the wavelet method, each one having its advantages and disadvantages, its preferred use for the removal of a specific type of artefacts and its application for filtering on specific problem types ^3, 24, 25, 28^. For example, PCA and ICA seem to be more effective for EOG artifact removal ^28^, while on their deficiencies PCA is not as powerful as ICA, but ICA requires more computational power for decomposition ^24^.

After clean signal data have been obtained, feature extraction follows to obtain essential features from the brain signals. Features are parameters providing information about the signal structure and it is in most cases a necessary step for an efficient EEG analysis. The types of features used in EEG analysis are mainly categorized into time-domain features (TDFs), frequency-domain features (FDFs) and time-frequency-domain features (TFDFs). The first, TDFs, are computed on raw, non-processed EEG signals or on preprocessed in the time domain ones. FDFs are calculated on the discrete-Fourier transform of raw EEG signals. Finally, TFDFs are determined on processed EEG signals that contain both time and frequency domain characteristics, such as short-time Fourier transform (STFT) or discrete wavelet transform (DWT) ^29^. There have been many techniques proposed in the literature for feature extraction from EEG signals. In 3, feature extraction methods have been analysed in three categories: spectral estimation methods, family of transforms, and time decomposition methods. In spectral estimation methods the Welch method is one of the most popular ones ^30^, while in the family of transforms methods ^31, 32^, Fourier transform (FT), STFT, continuous wavelet transform (CWT), DWT and wavelet packet decomposition (WPD) should be mentioned. In the time decomposition methods, empirical mode decomposition (EMD) ^33^ is the most characteristic example. Each method can be used for the computation of different features. In 34, fast Fourier transform (FFT) has been used for computing the normalized band power, while in 35, the gravity frequency, power percentage and frequency variability. DWT is also frequently used in many research papers for the computation of several statistical, wavelet, spectral and non-linear features, such as mean, median and standard deviation ^36, 37^, wavelet coefficients ^38, 39^, band power ^40^ and entropy ^40, 41^. In many cases, different bandpass filters are used for the decomposition of the signal into sub-bands and the extraction of features for them. Feature selection and dimensionality reduction are optional steps to improve the quality of results in case many features have arisen from the previous steps. In the study for epilepsy, for instance, the Kruskal Wallis test and the ANOVA test are encountered in the literature for feature selection ^42, 43^, and PCA, ICA, linear discriminant analysis (LDA), Laplacian eigenmaps (LE), kernel principal component analysis (KPCA) for dimensionality reduction. All features extracted are usually arranged into a vector, known as a feature vector.

Finally, the machine learning and testing phase involves mainly classification techniques aiming to classify the results into two or more classes. Indicatively, in epilepsy, researchers may want to classify the dataset instances to seizure/non-seizure ^40, 44^, or to control/interictal/ictal ^30, 36^. Different types of classifiers have been used by researchers in the field, including linear classifiers, such as support vector machine (SVM) ^42^, linear and logistic regression ^45, 46^ and LDA ^36, 47^, as well as artificial neural networks (ANN) based classifiers ^48– 50^, nearest neighbor classifiers, such as K-nearest neighbor (KNN) ^9, 51^ and decision trees and random forests ^4, 52^. In general, since there is a vivid and acute interest in the academic literature for EEG classification and analysis, all the primary methods and techniques in machine learning have been applied with diverse results based on their application field. Therefore, there is no straightforward way to conclude which are better techniques than others horizontally. Supervised learning methods are, however, considered in general more accurate than unsupervised alternative methods. Every method though has its advantages and disadvantages. For example, the ANN method provides high-accuracy results, but it is highly dependent on the number of neurons chosen in the hidden layer, of which their best number and combination is a result of a lot of trial and error. The KNN method is an easy-to-use and understandable method but requires careful and robust feature selection for high performance. After the application of the machine learning phase, the classification results are converted to decisions in the hands of physicians.

### 2.2 Actigraphies

According to 53, actigraphies are a valuable form of information, which can be used for estimating a range of sleep parameters and thus reveal some sleep disorders. Actigraphies have been proven to be an alternative solution to PSG due to their low-cost ^54^. Several machine learning approaches have been used for processing actigraphy data ^54^. Specifically, several sleep metrics can be computed through processing actigraphy files, which are mentioned briefly below:

- Time in bed (TIB)
- Sleep period
- Mean activity during TIB
- Sleep minutes during TIB
- Sleep onset latency
- Latency to persistent sleep
- Sleep efficiency
- Episodes of continuous sleep during TIB
- Sleep fragmentation index
- Wake after sleep onset

### 2.3 MRI

Several machine learning approaches have been introduced for supporting the diagnosis of dementia by using MRIs ^55– 57^. For processing the MRIs, several preprocessing steps are required, including motion correction, skull strip, white matter segmentation, tessellation, smooth1, inflate1, and so on. For instance, the work proposed in 58 used first recon-all from the FreeSurfer software package and then trained a deep learning model consisting of convolutional neural networks (CNNs).

Also, methods for measuring the brain cortical thickness have been introduced ^59^. This can be justified by the fact that assessing cortical brain properties and white matter atrophy has given complementary information about the Alzheimer’s disease (AD) process ^60^. Additionally, the authors in 61 used three-dimensional (3D) T1-weighted anatomical images and calculate the cortical thickness and volume. In terms of the preprocessing steps, the authors applied the following: brain extraction and intensity normalization, non-rigid registration n to a spherical template atlas, and implementation of the cortical parcellation exploiting the Desikan-Killiany atlas. For performing the experiments, the authors employed the FreeSurfer software package. Finally, a general linear model was used.

Structural equation modeling is also used as a statistical method and is exploited in 62 for teasing out the causal relationships among all these contributors to Alzheimer’s disease.

## 3 List of available tools

In this section, we describe the most common tools, which are used for performing all the experiments mentioned in Section 2.

**NumPy (RRID:SCR_008633).** NumPy is an open-source Python library ^63^. Several libraries are using NumPy, including scikit-learn, SciPy, statsmodels, etc. Below, we mention the benefits and limitations of this Python library:

- Benefits:
  – NumPy provides many functionalities, including indexing, broadcasting, or applying reduction operations. To be more precise, the user can have access to elements of a NumPy array via indexing, can apply multiplications or summations between arrays of different dimensions via broadcasting, and apply reduction operations, i.e., summation, mean, maximum, etc.
  – Statistical functions are provided through this library, including maximum, minimum, q-th percentile of the data, histograms, correlations, etc.
- Limitations:
  – NumPy does not offer many functionalities for pure statistical purposes. On the contrary, the statsmodels and SciPy libraries in Python offer many statistical functions.

**Pandas (RRID:SCR_018214).** Pandas is an open-source Python library used for working with structured datasets ^64^.

- Benefits:
  – In big data and especially medical data, datasets contain a lot of missing values. For performing statistical analysis and machine learning techniques, it is imperative that these missing values be imputed. The Pandas library offers functionalities for imputing missing data, including dropna and fillna. In terms of the fillna method, the user is able to apply some interpolation methods for propagating the values backward and forward.
  – The Pandas library provides the groupby function, where the user is capable of grouping the data by some identifiers and then applying statistical analyses. Also, the describe method is used, where the user can get descriptive statistics of all the columns and the rows of a dataset.
  – Similar to SQL, datasets can be combined, merged, and joined.
  – Correlation functions are provided through this library, including the Pearson correlation, Spearman rank correlation, and Kendall Tau correlation.
- Limitations:
  – As mentioned in the documentation, if one works with very large datasets and a tool like PostgreSQL fits the needs and requirements of the project, then this tool may be the right choice. Also, the documentation recommends the usage of other libraries, including Dask, which can operate more efficiently with larger datasets in parallel.
  – The methods for missing values imputation provided by this library are very simple. The sklearn library offers more algorithms for imputing missing values, including Nearest neighbors imputation and many more.

**DASK.** The DASK library is a Python library and is used for parallel computing ^65^. DASK comprises two parts, namely the dynamic task scheduling and the big data collections.

- Benefits:
  – DASK provides most of the functionalities offered by the NumPy library, including the reduction operations, arithmetic and scalar mathematics, slicing, and many more. Through the blocked algorithms, computations on arrays larger than memory can be performed.
  – DASK can use multiple threads or processes on a single machine, or a cluster of machines to process data in parallel.
  – The DASK DataFrame provides most of the utilities offered by the Pandas library, including the pearson correlation, drop duplicates, row-wise selections, etc.
- Limitations:
  – Some of the utilities offered by the NumPy library are not supported by DASK. Examples are operations like sort or tolist.
  – Some of the utilities offered by the Pandas library are not supported by DASK. To be more precise, setting a new index from an unsorted column is expensive and as a consequence performing operations like groupbyapply and join on unsorted columns is expensive too.

**SciPy (RRID:SCR_008058).** SciPy ^66^ constitutes an open-source Python library providing algorithms for many tasks, including optimization, integration, interpolation, differential equations, signal or image processing, etc. SciPy uses functions provided by the NumPy library and is being used by other libraries, such as scikit-learn.

- Benefits:
  – SciPy provides algorithms for signal processing, including convolutions, splines, filters, peak finding, spectral analysis, and many more. Through the fft module, the user is capable of applying the fast Fourier transform, including 1-D, 2, and N-D discrete Fourier transforms, the discrete cosine/sine transforms, and the fast Hankel transform.
  – Statistical functions are also provided by the SciPy library through the stats module. Specifically, one can apply statistical tests, including the t-test, Mann-Whitney U rank test, Wilcoxon signed-rank test, Kruskal-Wallis H-test, etc. Also, one can plot cumulative frequency histograms and compute summary statistics, i.e., mean, variance, skewness, kurtosis, etc. In terms of the correlation functions, the point-biserial, the Spearman, and the Pearson are provided.
  – In terms of the image processing, one can use the module *ndimage* and perform linear and non-linear filtering, region labelling and processing, B-spline interpolation, etc.
- Limitations:
  – Although Scipy provides implementations of statistical tests, it does not provide the functionality of the adjustment/correction of the p-values through multiple comparisons. On the contrary, this functionality is provided by the statsmodels library.

**Sklearn (RRID:SCR_019053).** Scikit-learn is an open-source Python library ^67^ offering many functionalities of machine learning techniques, clustering, preprocessing data, and more.

- Benefits:
  – The scikit-learn library provides methods of missing data imputation, including univariate-multivariate imputation, nearest neighbours imputation, etc.
  – Functionalities for clustering and dimensionality reduction techniques are also provided, including the principal component analysis (PCA), t-SNE, etc. These methods are also used for visualization purposes.
- Limitations:
  – Despite the fact that scikit-learn provides methods, which can be used for neuroimaging and time-series data, there are libraries, including Nilearn, MNE, etc. which provide more functionalities.

**statsmodel (RRID:SCR_016074).** Statsmodels is a library for statistical and econometric analysis in Python ^68^.

- Benefits:
  – This library provides access to time-series analysis, including univariate autoregressive models (AR), vector autoregressive models (VAR), and univariate autoregressive moving average models (ARMA). These methods can be used for time-series forecasting. Also, autocorrelation, partial autocorrelation, periodogram, etc. are supported.
  – Compared with the SciPy library, the statsmodels library implements more statistical packages. Also, methods for the adjustment/correction of the p-values with multiple tests are also supported, including the Bonferroni, Benjamini-Hochberg ^69^, Simes-Hochberg, Holm-Sidak, etc.
  – Missing values imputation methods are also supported, including the multiple imputation with chained equations (MICE), and the Bayesian imputation using a Gaussian model.
- Limitations:
  – Some of the functionalities provided via the SciPy library are not available in the statsmodels library. For instance, multidimensional image processing, peak finding, or spectral analysis of signals are some of these methods.

**pmdarima.** pmdarima is a Python library providing implementations of ARIMA estimators to the user ^70^.

- Benefits:
  – The most important contribution of the pmdarima library is the implementation of the AutoArima method, which automatically discovers the optimal order for an ARIMA model. It is the implementation of the R programming language.
  – pmdarima provides implementations of the ARIMA estimator and statistical tests, including OCSB test of seasonality, CH test for seasonality, KPSS test for stationarity, PP test for stationarity, and ADF test for stationarity.
  – Also, pmdarima provides a number of transformer classes for pre-processing time series or exogenous arrays, including the Box-Cox transformation and the log transformation.
- Limitations:
  – The statsmodels library implements more statistical tests. However, one should not overlook the implementation of the AutoArima.

**sktime.** sktime constitutes a scikit-learn compatible Python library with a unified interface for machine learning with time series ^71, 72^.

- Benefits:
  – The sktime library provides algorithms for time-series forecasting, annotation, classification, regression, clustering, and transformations.
  – Regarding time-series forecasting, reduction forecasters that use sklearn regressors or sktime time series regressors to make forecasts, exponential smoothing based forecasters, AR(I)MA type forecasters, ensembles, stacking, etc. are supported by the library.
  – In terms of clustering, clusteing models are provided, including time series K-mean implementation, time series K-medoids implementation, K-shape algorithm wrapper tslearns implementation, and Kernel algorithm wrapper tslearns implementation.
- Limitations:
  – The time series annotation API is still experimental.

**Pingouin (RRID:SCR_022261).** Pingouin is an open-source Python library, which provides implementations of statistical tests and is based on Pandas and NumPy ^73^.

- Benefits:
  – Statistical tests are supported, including ANOVA, t-test, bayes factors, multivariate tests, and many more. Also, the functionality of correcting the p-values is supported.
  – The t-test function of Pingouin returns the T-value, the p-value, the degrees of freedom, the effect size (Cohen’s d), the 95% confidence intervals of the difference in means, the statistical power and the Bayes factor (BF10) of the test. On the contrary, the SciPy library returns only the T-value and the p-value.
- Limitations:
  – The statsmodels implements more statistical tests.

**Nitime (RRID:SCR_002504).** Nitime is a Python library and is being used for neuroimaging data (especially functional magnetic resonance imaging data) ^74^.

- Benefits:
  – The nitime library provides the module called algorithms. This module provides implementations of spectral transforms, coherency, regularized coherency, and event-related analysis.
  – For instance, in terms of spectral transforms the calculation of a standard periodogram and cross-spectral density estimates based on both the regular and multi-taper periodograms are provided.
  – In terms of coherency, this technique is used in order to calculate the functional connectivity between time-series derived from different voxels in the brain, or different ROIs.
  – With regards to the event-related analysis, univariate statistics, which are calculated separately for the time-series in each voxel or each ROI, are provided.
- Limitations:
  – The nitime library provides limited support for preprocessing the data.

**MNE software (RRID:SCR_005972).** MNE constitutes an open-source Python package for exploring, visualizing, and analyzing human neurophysiological data: magnetoencephalography (MEG), EEG, stereoelectroencephalography (sEEG), electrocorticography (ECoG), near-infrared spectroscopy (NIRS), and more ^75^.

- Benefits:
  – The MNE library provides many functions for reading data in several formats, including BrainVision (.vhdr, .vmrk, .eeg), European data format (.edf), BioSemi data format (.bdf), general data format (.gdf), Neuroscan CNT (.cnt), and more.
  – Methods for processing data are provided, including filtering (notch), detection and removal of artifacts (ICA and SSP), spectral analysis, making montages, etc.
  – The MNE library provides plots of data.
  – In terms of MRI processing, the MNE library implements some methods, including the coregistration for subjects with structural MRI.
- Limitations:
  – The MNE library provides limited support for MRIs in comparison with other libraries.

**YASA.** Yet Another Spindle Algorithm (YASA) is a sleep analysis toolbox in Python ^76^.

- Benefits:
  – This library provides the functionality of the automatic sleep stating of polysomnography data. Also, it provides methods for event detection. Such events are the following: sleep spindles, slow-waves, rapid eye movements on single or multi-channel EEG data.
  – The library supports also methods for artifact rejection, spectral analyses, including bandpower, IRASA method, spectrogram, overnight coupling between EEG bandpower and heart rate variability, and many more.
  – Finally, YASA provides hypnogram analysis, i.e., sleep statistics and stage transitions.
- Limitations:
  – MNE can be used for reading data and preprocessing stages.

**WONAMBI ( https://wonambi-python.github.io/).** WONAMBI is a package used for the analysis of EEG, ECoG and other electrophysiology modalities.

- Benefits:
  – This library provides the functionality of reading files of the following formats: Axon (.abf, ABF2 only), Brain Vision (.vhdr, .vmrk, .eeg / .dat), EEGLAB (.set, .set / .fdt), European data format (.edf), etc.
  – An interface for sleep scoring is also supported.
  – Computes frequency analysis (spectrogram), time-frequency analysis (short-time spectrogram, Morlet wavelet), and detects spindles and slow waves.
- Limitations:
  – YASA offers more functionalities than WONAMBI.

**SleepPy.** SleepPy constitutes an open source python package prividing functions for the assessment of sleep quantity and quality ^77^.

- Benefits:
  – This library can process multi-day streams of raw accelerometer data (X, Y & Z) from wrist-worn wearable devices. Sleep reports and visualizations for each recording day are produced.
  – This package derives activity index, identifies major rest period, performs sleep/wake classification, and calculates sleep measures.
- Limitations:
  – SleepPy can work with data generated by the GeneActiv wrist-worn devices (GeneActiv.bin files). It can also support .csv files generated by GeneActiv’s software.

**Visbrain.** Visbrain is an open-source Python package used for the visualization of the brain signals ^78^.

- Benefits:
  – This package provides the *Signal* module, which implements functionalities, such as de-trending and demeaning, filtering (lowpass, highpass, bandpass, bandstop), extracting the amplitude, phase or power in specific frequency bands.
  – The *Sleep* module provides time-frequency methods, including the fourier-based spectrogram, the morlet’s wavelet, and the multitaper-based Wigner spectrogram.
  – Detection of events is also supported, including sleep spindles, K-complexes, rapid-eye movements, slow waves, muscle twitches, and peaks.
  – The *Brain* module provides interface with many functionalities, including connecting deep sources, adding time series, pictures, region of interest, etc.
- Limitations:
  – Visbrain does not provide data analysis functions, which are provided by other libraries.

**Neuropycon.** Neuropycon is a Python open-source multi-modal brain data analysis toolkit ^79^. It includes two different packages, namely the ephypype and the graphpype.

- Benefits:
  – The ephypype package is based on the MNE library and provides pipelines and analyses for electrophysiology data. Specifically, one can apply the ICA algorithm for removing the eyes and heart related artifacts, can compute the power spectral density, the inverse solution starting from raw data, and perform connectivity analysis in sensor or source space.
  – The graphpype package includes graph-theoretical tools for connectivity matrices. In addition, the pipeline called *inv_ts_to_graph* is capable of running the spectral connectivity and the graph computation over time series. Also, the computation of connectivity matrices from preprocessed functional MRI is supported.
- Limitations:
  – Difficulties pertaining to the creation of the workflows.

**NiLearn (RRID:SCR_001362).** Nilearn constitutes a Python library used for neuroimaging data and provides both statistical and machine learning tools ^80^.

- Benefits:
  – Nilearn provides the implementation of generalized linear models accompanied with examples in terms of both the first level and second level analysis. Also functionalities for manipulating brain image volumes are supported, including smoothing an image, comparing the means of two images, breaking an atlas of labels in separated regions, resampling an image to a template, etc.
  – Advanced statistical analysis of brain images is supported, including advanced decoding using scikit-learn, multivariate decomposition, and many more.
  – Nilearn provides functionalities for visualizing MRI data in an interactive way.
- Limitations:
  – Nilearn does not support functionalities for reading MRI data.

**Dipy (RRID:SCR_000029).** Diffusion imaging in Python (Dipy) is an open-source Python library used for the analysis of diffusion magnetic resonance imaging (dMRI) data ^81^.

- Benefits:
  – DIPY provides algorithms for statistical analysis, reconstruction, registration, tractography, denoising, visualization, and preprocessing.
  – Several methods are available for denoising, including Patch2Self ^82^, Non-Local Means, local PCA via empirical thresholds, Marcenko-Pastur PCA algorithm, and the supression of Gibbs artifacts.
  – With regards to the registration, the following methods are supported: Affine Registration in 3D, Affine Registration with Masks, Symmetric Diffeomorphic Registration in 2D, Symmetric Diffeomorphic Registration in 3D, Diffeomorphic Registration with binary and fuzzy images, and Streamline-based Registration.
- Limitations:
  – No significant limitations.

**Antropy.** AntroPy is a Python 3 package providing several time-efficient algorithms for computing the complexity of time-series. It can be used for example to extract features from EEG signals.

- Benefits:
  – This library provides many algorithms, including Approximate Entropy, Hjorth mobility and complexity, Number of zero-crossings, Permutation Entropy, Sample Entropy, Spectral Entropy, Singular Value Decomposition entropy, Detrended fluctuation analysis (DFA), Higuchi Fractal Dimension, Katz Fractal Dimension, and Petrosian fractal dimension.
- Limitations:
  – As mentioned in the documentation, the results need to be double-checked.

**UK Biobank accelerometer analysis.** UK biobank accelerometer analysis is a tool used for the extraction of information from large accelerometer datasets. It can be installed via Python and also needs Java to be installed ^83– 86^.

- Benefits:
  – This package can process data from raw GENEActiv. Bin files, raw Actigraph .gt3x files (both versions 1 and 2), raw gzipped CSV files. Moreover, this package provides the opportunity for processing hundreds of accelerometer files.
- Limitations:
  – Limited support for various data formats.

**PyEEG.** PyEEG is an open-source Python library providing functionalities for extracting features from EEG data ^87^.

- Benefits:
  – Many features can be computed, including the Power Spectral Intensity (PSI) and Relative Intensity Ratio (RIR), Petrosian Fractal Dimension (PFD), Higuchi Fractal Dimension (HFD), Hjorth mobility and complexity, Spectral Entropy (Shannon’s entropy of RIRs), SVD Entropy, etc
- Limitations:
  – PyEEG does not provide support for reading data.

**Eeglib.** eeglib is an open-source Python library used for feature extraction from EEG signals and is based on sliding windows ^88^.

- Benefits:
  – This library can read data from three formats, namely csv, EDF, and NumPy arrays.
  – It provides functionalities of bandpass filter, ICA, and z-scores normalization to the raw EEG data.
  – FFT, DWT, Power Spectral Density, Band Power, Hjorth Parameters, Detrented Fluctuation Analysis, Sample Entropy, and many more are supported.
- Limitations:
  – MNE can import data in more file formats than Eeglib.

**NiBabel (RRID:SCR_002498).** Nibabel is a Python library used for reading neuroimaging data ^89^.

- Benefits:
  Nibabel provides access to the most common medical and neuroimaging file formats, including ANALYZE (plain, SPM99, SPM2 and later), GIFTI, NIfTI1, NIfTI2, MINC1, MINC2, MGH and ECAT as well as Philips PAR/REC.
  – Access to head information and the image data is supported.
  – Easy-to-read documentation with many examples.
- Limitations:
  – Limited support for DICOM.

**AcqKnowledge Software (RRID:SCR_014279) ( https://www.biopac.com/)**

- Benefits:
  – Offers integrated functionality to control the data acquisition process using the associated hardware device.
  – Reports and displays all data gathered by actigraphy devices and displays sleep and actigraphy metrics.
  – Has many display options and analytics functions, relating to both actigraphies and eeg data.
  – Automated analysis for ECG, HRV, EEG, EMG, EGG, and many more.
- Limitations:
  – Proprietary application designed to work in tandem with specific devices

**MotionWare (RRID:SCR_022253) ^90^**

- Benefits:
  – Reports and displays all data gathered by actigraphy devices and offer sleep and actigraphy analytics, including automatic sleep scoring, circadian rhythm NPCRA analysis and physical activity analysis.
  – Offers detailed view and plot options.
  – Has cloud integration for the handling of data.
- Limitations:
  – Proprietary application designed to work in tandem with specific devices.

**Respironics Actiware (RRID:SCR_016440) ^91^**

- Benefits:
  – Reports and displays all data gathered by actigraphy devices and offer sleep and actigraphy analytics, including automatic sleep scoring.
  – Offers detailed view and plot options.
- Limitations:
  – Proprietary application designed to work in tandem with specific devices (Actiwatch), but it at least supports using data from older legacy versions of the devices.
  – Actiwatch, the associated device has been reportedly recently discontinued and new ones aren’t offered in clinician or research markets, making the future and usability of Actiware uncertain ^92^.

**MedPy ( http://loli.github.io/medpy/).**

- Benefits:
  – Open-source application with code available in public code repositories.
  – Can handle many different formats of medical files including large files with multidimensional features.
  – Offers several two dimensional image filters in a n-dimensional version appropriate for medical imaging.
  – Offers image feature extractors such as histograms, means, indices.
  – Can create DIMACS graphs from sources by using aribtrary energy n-dimensional images.
- Limitations:
  – No major release the last years but fixes and minor development seems to continue to this day.
  – Its a library focused on specific and bare bone functionality that needs effort from non technical users to implement in applications.

**Skimage.** scikit-image is a collection of algorithms for image processing ^93^.

- Benefits:
  – This package provides algorithms for the detection of edges and lines, geometrical transformations and registration, filtering and restoration, detection of features and objects, segmentation of objects, and more.
- Limitations:
  – Skimage does not provide many functionalities for medical images.

**OpenCV (RRID:SCR_015526).** OpenCV is a library available in C++, Java, and Python and is used for image processing ^94^.

- Benefits:
  – OpenCV provides many functionalities, including image filtering, feature and object detection, image segmentation, motion analysis, object tracking, Canny edge detector, Hough Line/Circle Transform, and Affine Transformations, histogram equalisation, calculation, and comparison.
  – OpenCV provides a forum, where one could seek for help.
- Limitations:
  – Compared to other libraries, i.e., Nilearn, this library offers general functions.

**fMRIPrep (RRID:SCR_016216).** Fmriprep is a functional magnetic resonance imaging (fMRI) data preprocessing pipeline ^95^.

- Benefits:
  – The fMRIPrep pipeline uses a combination of tools from well-known software packages, including FSL, ANTs, FreeSurfer and AFNI.
  – Optimal data processing quality is achieved, preprocessing quality reports are generated, processing steps are automated.
- Limitations:
  – Fmriprep is not easily customizable, meaning that changes to the steps of a pipeline may be difficult.

**Nipype (RRID:SCR_002502).** Nipype is a Python project providing interface to existing neurimaging softwares ^96^.

- Benefits:
  – Nipype provides interfaces for many tools, including AFNI, ANTs, dcm2nii, DIPY, FreeSurfer, FSL, MIPAV, MNE, Statistical Parametric Mapping (SPM), 3D Slicer, etc.
  – Nipype provides a forum, where one can seek for assistance.
- Limitations:
  – Although it provides access to neuroimaging tools, it does not support all the functionalities offered by these tools. For instance, SAMSEG of FreeSurfer is not supported via Nipype.
  – One can use FreeSurfer, instead of the interface of nipype, since FreeSurfer is dockerized.

**NLTools.** NLTools is a Python package for analyzing neuroimaging data ^97^.

- Benefits:
  – NLTools provides a wide number of functionalities, including the identification of spikes from time-series data, getting the median of each voxel or image, applying Fisher’s r to z transformation, extracting brain connected regions into separate regions, running a mass-univariate regression across voxels, applying spatial smoothing, and many more.
- Limitations:
  – No significant limitations.

**ActiLife.** ActiLife is ActiGraph’s premier actigraphy data analysis software platform. ^98^

- Benefits:
  – Developed specifically for advanced sleep research and clinical applications
  – Includes a variety of sophisticated sleep analysis and reporting tools.
  – Representation of graphical sleep/wake activity and calculate sleep statistics such as onset, sleep latency, amount of sleep, and sleep efficiency using several validated scoring algorithms
- Limitations:
  – The full functionality of the software requires activation licenses.

**PyActigraphy.** PyActigraphy constitutes an open-source Python library used for actigraphy data visualization and analysis ^99^.

- Benefits:
  – PyActigraphy can read data from many formats, including:
    * Actigraph: wGT3X-BT
    * CamNtech: Actiwatch 4 and MotionWatch 8
    * Condor Instrument: ActTrust 2
    * Daqtix: Daqtometer
    * Respironics: Actiwatch 2 and Actiwatch Spectrum (plus)
    * Tempatilumi (CE Brasil)
  – Algorithms for detecting resting periods are supported, including the Cole-Kripke’s ^100^, Sadeh’s ^101^, Crespo’s ^102^, and Roenne-berg’s ^103^.
  – Advanced signal processing functions are provided, including the Cosinor Analysis ^104^, Detrended Fluctuation Analysis ^105, 106^, Functional Linear Modelling ^107^, Locomotor Inactivity during sleep ^108^, Singular Spectrum Analysis ^109^.
- Limitations:
  – PyActigraphy package is currently under development and many more features are expected to be implemented.

**FactorAnalyzer ( https://factor-analyzer):** FactorAnalyzer constitutes an open-source Python library used for performing exploratory factor analysis (EFA) and confirmatory factor analysis (CFA).

- Benefits:
  – Includes class to perform exploratory and factor analysis, with several optional rotations.
  – Includes class to perform confirmatory factor analysis, with certain pre-defined constraints.
  – In exploratory factor analysis, factor extraction can be performed using a variety of estimation techniques.
- Limitations:
  – The Confirmatory Factor Analyzer class is very experimental at this point.

**lifelines:** lifelines constitutes an open-source Python library used as a complete survival analysis library ^110^.

- Benefits:
  – Incorporates the most popular parametric, semi-parametric and non-parametric models
  – Can handle right, left and interval censored data
  – Provides internal plotting methods
  – Facilitates an easy and intuitive way of use
- Limitations:
  – No significant limitations.

**zEpid ( https://zepid):** zEpid constitutes an open-source Python toolkit used for epidemiology analysis.

– Benefits:
  – Provides basic epidemiology calculations and summary measure calculations from summary data
  – Supports several different graphics generated using matplotlib.
  – Provides sensitivity analyses to determine the robustness of findings against certain assumptions or unmeasured factors.
Limitations:
  – No significant limitations.

**semopy:** semopy (Structural Equation Models Optimization in Python) is designed to help statisticians that employ SEM techniques to handle their research using Python ^111, 112^.

- Benefits:
  – Provides a User-friendly syntax for specifying SEM models.
  – Provides Random Effects model (or Linear Mixed Models/LMM) with SEM to take population structure into account when necessary.
  – Supports the prediction of data, the imputation of missing values and the estimation of factor scores.
  – Incorporates some utilities to perform EFA to extract measurement structure from the data.
  – Supports random models generation to test semopy or other SEM packages features
  – Excellent performance when applied to big datasets.
  – Active community with issue tracking and updates
- Limitations:
  – No significant limitations.

**lavaan:** The lavaan package is developed to provide users, researchers and teachers a free open-source, but commercialquality package for latent variable modeling ^113^.

- Benefits:
  – Provides the estimation of large variety of multivariate statistical models, including path analysis, confirmatory factor analysis, structural equation modeling and growth curve models
  – It is deemed as reliable, open and extensible
  – Facilitates an easy and intuitive way of use
  – Active community with issue tracking and frequent new releases
- Limitations:
  – No significant limitations.

**scikit-survival:** scikit-survival is a Python module for survival analysis built on top of scikit-learn ^114^.

- Benefits:
  – Enhance understanding of predictions in Survival Analysis
  – Provides evaluating Survival Models, Gradient Boosted and Penalized Cox Models
  – Active community with issue tracking and frequent new releases
- Limitations:
  – No significant limitations.

**EPViz:** EPViz (EEG Prediction Visualizer) is a tool to aid researchers in developing, validating, and reporting their predictive modeling outputs ^115^.

- Benefits:
  – Provides a basic functionality for EEG signals visualisation, with average reference and longitudinal bipolar montages and an annotation editor
  – Allows researchers to load a PyTorch deep learning model.
  – Computing basic signal statistics
  – Incorporates spectrogram computation and analysis
- Limitations:
  – Simple signal viewer good for research purposes, but lacks the needed resolution for clinical diagnosis.

**EEGraph:** EEGraph is a Python library to model electroencephalograms (EEGs) as graphs, so the connectivity between different brain areas could be analyzed ^116^.

- Benefits:
  – Incorporates several different connectivity measures in EEGraph, like correlations, coherence, WPLI, PLV, etc.
  – Supports the export of the graph as a NetworkX graph-like object or/and as graphically visualised object.
- Limitations:
  – No significant limitations.

**EDFbrowser ( https://www.teuniz.net/):**

- Benefits:
  – EDFBrowser offers many visualisation options, such as filtering, saving montages with all the parameterisation, including the option to calibrate according the users screen and set the timescale of EDF channels per centimeters of the screen, useful for many neurologists, a functionality not usually present in most open source tools.
  – Allows annotation of file, combined with storing and displaying hypnograms in annotation form.
  – Provides many analysis functions such as power spectral analysis, color density spectral array, amplitude integrated electroencephalography, waveform averaging ^117^.
  – Offers capabilities to repair, edit or anonymise files.
- Limitations:
  – While remote control capabilities exist through a direct TCP connect, very few options are available.

**FreeSurfer (RRID:SCR_001847) ( https://surfer.nmr.mgh.harvard.edu/):** FreeSurfer is an open source neuroimaging toolkit for processing, analysing, and visualising human brain MR images.

- Benefits:
  – FreeSurfer can be used for the analysis and visualisaton of structural, functional, and diffusion neuroimaging data from cross-sectional and longitudinal studies.
  – FreeSurfer provides the algorithm of recon-all, which includes 31 processing stages, including motion correction and conform, skull strip, spherical mapping/registration, and many more.
  – FreeSurfer supports the functionality of SAMSEG, which is a tool to robustly segment dozens of brain structures from head MRI scans without preprocessing.
- Limitations:
  – The running time of recon-all may range from some hours to days.

**Neurodesk (RRID:SCR_023714) ( https://www.neurodesk.org/)**: NeuroDesk constitutes a flexible, scalable and browser-based data analysis environment for reproducible neuroimaging.

– Benefits:
  – NeuroDesk provides analyses of electrophysiological and neuroimaging data, including functional imaging, hippocampus, image reconstruction/registration/segmentation, structural imaging, spine, statistics, etc.
  – With regards to the electrophysiological data, NeuroDesk provides access to brainstorm, eeglab, fieldtrip, and MNE.
  – Regarding neuroimaging data, the FreeSurfer is supported. All the analyses supported by FreeSurfer, including visualisation approaches, are provided in NeuroDesk.
  – 3D Slicer is also supported.
  – Documentation for using docker containers is supported.
  – NeuroDesk applications can be also exploited in Google Colab.
Limitations:
  – No significant limitations

## 4 Conclusions

In contrast with other studies, which examined some methods and tools used for supporting the diagnosis of one disorder, this study reviewed advanced analytics and ML methods for neurocognitive disorders (dementia), sleep disorders, and epilepsy. Additionally, this is the first study reviewing all the available tools, which can be used for processing different types of data, including PSG, actigraphies, MRIs, and simple tabular data (questionnaire data). At the same time, all the available tools are accompanied by some benefits and limitations. We hope that this current study will be helpful for AI and ML researchers working towards the diagnosis of complex brain disorders, including dementia, epilepsy, and sleep disorders. Additionally, these methods and tools can be used for other brain disorders as well using actigraphies, PSG, and MRIs.

## Ethics and consent statement

Ethical approval and consent were not required.

## Data Availability

No data are associated with this article.
