# Supplementary material for: Overview of methods and available tools used in complex brain disorders
Source: Open Res Eur. 2023 Sep 19;3:152. [Version 1] doi: 10.12688/openreseurope.16244.1 (PMC10882203; doi:10.12688/openreseurope.16244.1)
Supplement: Peer review history

# Reviewer report: Reviewer response for version 1

**DOI:** 10.21956/openreseurope.17538.r36449

**Published:** 2024-02-21

**Authors:** Euripidis Loukis

**Affiliations:** 1 University of the Aegean, Samos, Greece

This article provides a comprehensive review of the methods and tools used for supporting the diagnosis of complex brain disorders. Initially it reviews the main methods used for this purpose: polysomnography, actigraphies and MRI. Then it reviews a large number of software tools (libraries) that can be used for the analysis of relevant signals. The authors seem to have a good knowledge of these topics, and the article is quite informative, as it includes a lot of useful information.

 However, the article could greatly benefit from some improvements. The most important of them might be the restructuring of section 3, which now includes a lot of quite heterogeneous software tools (libraries), which are not grouped into homogeneous sections: generic tools for statistical processing, more specialised tools for neuroimaging data, for EEG signals, for data from artigraphy devices; also, it includes irrelevant tools that do not concern the diagnosis of mental disorders, such as the ones in pages 12 and 13 that concern survival analysis, epidemiology analysis, SEM, latent variable modeling and predictive modeling (it would be better if these were not included). So it would be good to group these tools in separate sub-sections of section 3.

 Also, this article lacks a methodology section, which might describe how these tools have been identified and selected for inclusion, and also how the literature review presented in section 2 ('Related work') was conducted (how the papers on which this review was based were identified - probably through a search in one or more of the well established papers databases using some criteria). So it would be good to include a methodology section (even a short one).

 Furthermore, this literature review of section 2 is 'asymmetric': while sub-section 2.1 about polysomnography is quite detailed (having a length of 2,5 page columns), section 2.2 about actigraphies and section 2.3 about MRI are much less detailed (having a length of about 0,5 page column each). So it would be good to increase 2.2 and 2.3 and include similar elements and details with the ones of 2.1.

 Finally, the article includes a lot of useful information, as mentioned above, but seems inconclusive - the final conclusions section 4 is quite short. So, it would be good to include more the conclusions that are drawn from the extensive information included in the previous sections.

 I believe that the above improvements can be easily done, as the authors seem to have a good knowledge of this area, leading to a substantial improvement of the quality of this quite interesting article

Is the review written in accessible language?

Yes

Are all factual statements correct and adequately supported by citations?

Yes

Are the conclusions drawn appropriate in the context of the current research literature?

Yes

Is the topic of the review discussed comprehensively in the context of the current literature?

Yes

Reviewer Expertise:

One of my research areas are medical information systems and decision support systems, with main emphasis on systems using advanced medical data processing methods, such as machine learning ones - in general I am conducting research in the wider area of information systems and decision support systems

I confirm that I have read this submission and believe that I have an appropriate level of expertise to confirm that it is of an acceptable scientific standard.

# Reviewer report: Reviewer response for version 1

**DOI:** 10.21956/openreseurope.17538.r37162

**Published:** 2024-02-15

**Authors:** George Tsihrintzis

**Affiliations:** 1 University of Piraeus, Piraeus, Greece

In this work, the authors investigate the available literature on complex brain disorders in a comprehensive way. The authors survey includes software tools that are currently available towards processing multimodal data to diagnose and classify complex brain disorders in the population. The text also includes a presentation of the limitations of the various available tools, which is particularly useful in identifying targets for future research and development.

 Clearly, the manuscript topic is of particular importance to society, as people live longer and exhibit complex brain disorders more often and more seriously.

 The manuscript is very well-organized and written.

 A suggestion that may improve the article is to include a (perhaps short) discussion on the potential usefulness of generative AI technologies as assistive tools towards the diagnosis of complex brain disorder.

Is the review written in accessible language?

Yes

Are all factual statements correct and adequately supported by citations?

Yes

Are the conclusions drawn appropriate in the context of the current research literature?

Yes

Is the topic of the review discussed comprehensively in the context of the current literature?

Yes

Reviewer Expertise:

Pattern Recognition, Machine Learning, Data Analytics, AI-empowered Software

I confirm that I have read this submission and believe that I have an appropriate level of expertise to confirm that it is of an acceptable scientific standard.
